# Effect of pediatric ventilation weaning technique on work of breathing

**DOI:** 10.1186/s12931-022-02106-6

**Published:** 2022-07-13

**Authors:** Jefta van Dijk, Alette A. Koopman, Limme B. de Langen, Sandra Dijkstra, Johannes G. M. Burgerhof, Robert G. T. Blokpoel, Martin C. J. Kneyber

**Affiliations:** 1 Division of Paediatric Critical Care Medicine, Department of Paediatrics, Beatrix Children’s Hospital, University Medical Center Groningen, University of Groningen, Internal Postal Code CA 62, P.O. Box 30.001, 9700 RB Groningen, The Netherlands; 2 Department of Epidemiology, University Medical Center Groningen, The University of Groningen, Groningen, The Netherlands; 3 Critical Care, Anaesthesiology, Peri-Operative and Emergency Medicine (CAPE), University of Groningen, Groningen, The Netherlands

**Keywords:** Pediatrics, Mechanical ventilation, Work of breathing, Weaning, Pressure–rate-products, Pressure–time-product, Phase angle

## Abstract

**Background:**

Ventilator liberation is one of the most challenging aspects in patients with respiratory failure. Most patients are weaned through a transition from full to partial respiratory support, whereas some advocate using a continuous spontaneous ventilation (CSV). However, there is little scientific evidence supporting the practice of pediatric ventilator liberation, including the timing of onset of and the approach to weaning mode. We sought to explore differences in patient effort between a pressure controlled continuous mode of ventilation (PC-CMV) [in this cohort PC assist/control (PC-A/C)] with a reduced ventilator rate and CSV, and to study changes in patient effort with decreasing PS.

**Methods:**

In this prospective physiology cross-over study, we randomized children < 5 years to first PC-A/C with a 25% reduction in ventilator rate, or CSV (continuous positive airway pressure [CPAP] + PS). Patients were then crossed over to the other arm. Patient effort was measured by calculating inspiratory work of breathing (WOB) using the Campbell diagram (WOB_Campbell_), and by pressure–rate-product (PRP) and pressure–time-product (PTP). Respiratory inductance plethysmography (RIP) was used to calculate the phase angle. Measurements were obtained at baseline, during PC-A/C and CPAP + PS, and during decreasing set PS (maximum -6 cmH_2_O).

**Results:**

Thirty-six subjects with a median age of 4.4 (IQR 1.5–11.9) months and median ventilation time of 4.9 (IQR 3.4–7.0) days were included. Nearly all patients (94.4%) were admitted with primary respiratory failure. WOB_Campbell_ during baseline [0.67 (IQR 0.38–1.07) Joules/L] did not differ between CSV [0.49 (IQR 0.17–0.83) Joules/L] or PC-A/C [0.47 (IQR 0.17–1.15) Joules/L]. Neither PRP, PTP, ∆Pes nor phase angle was different between the two ventilator modes. Reducing pressure support resulted in a statistically significant increase in patient effort, albeit that these differences were clinically negligible.

**Conclusions:**

Patient effort during pediatric ventilation liberation was not increased when patients were in a CSV mode of ventilation compared to a ventilator mode with a ventilator back-up rate. Reducing the level of PS did not lead to clinically relevant increases in patient effort. These data may aid in a better approach to pediatric ventilation liberation.

*Trial registration* clinicaltrials.gov NCT05254691. Registered 24 February 2022

**Supplementary Information:**

The online version contains supplementary material available at 10.1186/s12931-022-02106-6.

## Background

Mechanical ventilation (MV) is one of the core features of the pediatric intensive care unit (PICU). Despite lifesaving, MV is also associated with undesired effects, which may ultimately affect physical functioning and quality of life. These include amongst others the occurrence of ventilator induced lung injury (VILI), nosocomial pneumonia, upper airway trauma, hemodynamic instability and increased need for sedation or even neuromuscular blockade with subsequent risk for withdrawal syndrome or delirium [1–3]. This underscores the need to start ventilation liberation as soon as the clinical condition of the patient allows for this. It is estimated that almost half of the total ventilation time is related to weaning [4, 5]. Unfortunately, there is little scientific evidence supporting the practice of pediatric ventilation liberation, including the timing of onset of and the approach to weaning. This can be partly explained by the relative short ventilation time and low extubation failure rates observed in the pediatric population [6–8].

The most common approach to weaning in infants and children is a gradual reduction of ventilatory support through a reduction of the ventilator rate and/or a reduction in inspiratory pressures when the patient is in pressure controlled mode of ventilation (PCV) [9]. Alternatively, it has also been proposed to periodically use a continuous spontaneous ventilation (CSV) mode (i.e., pressure support [PS]) in combination with continuous positive airway pressure (CPAP) and alternate this with complete ventilatory support. The rationale for this approach is to (slowly) train and reactivate the respiratory muscles [9]. However, there is no pediatric data that has shown superiority of one approach over the other [10]. Aside from weaning technique, the unanswered question is also how much PS to give. Both over- and undersupport may exert negative effects on respiratory muscle function and patient effort.

Irrespective of the approach chosen by the clinical team, it is imperative to assess work of breathing (WOB) when the patient is weaned from the ventilator. The gold standard for measuring inspiratory WOB is through the Campbell diagram (WOB_CAMPBELL_) by making use of an esophageal catheter. This diagram reflects the energy that is needed to expand the lungs and chest wall during inspiration [11]. Surrogate parameters include esophageal pressure swing (∆Pes), the pressure rate product (PRP) and the pressure time product (PTP), which both can distinguish patient effort from the total effort, and the phase angle calculated from respiratory inductance plethysmography readings [12–15].

Based on the hypothesis that weaning using CSV would not result in increased WOB, irrespective of the level of PS, we sought to characterize in a randomized cross-over trial patient effort during ventilator weaning by comparing WOB_CAMPBELL_, PTP, PRP, ∆Pes and the phase angle measured during PC-A/C with a reduced ventilator rate and during CSV in ventilated children who were deemed eligible for weaning by the attending physician. We also studied if there was a relationship between patient effort and the level of PS.

## Methods

### Study design

This study was designed as a prospective, physiological, randomized cross-over study comparing two different weaning strategies and the effect of the level of PS on the work of breathing in mechanically ventilated children admitted to the 20-bed tertiary medical-surgical pediatric intensive care unit (PICU) of the Beatrix Children’s Hospital, University Medical Center Groningen (Groningen, The Netherlands). The study was approved by the institutional review board (IRB) (NL38361.042.11), and written informed consent was obtained from parents or legal caretakers.

### Patients

Patients were daily assessed for eligibility when the attending physician who identified the patient ability for weaning, which was defined by the ability to maintain adequate oxygenation and ventilation under stable ventilator settings (i.e., no need for increase of inspiratory pressures or positive end-expiratory pressure, and fraction inspired oxygen (FiO_2_) < 0.5 within 6 h prior to enrolment). Subjects were enrolled if they were younger than 5 years of age, ventilated for at least 24 h, able to trigger the ventilator and had sufficient respiratory drive and stable hemodynamics (i.e., no need for increase in vaso-active drugs and/or fluid challenges at least 6 h prior to enrolment). Excluded were subjects born prematurely with a corrected gestational age < 40 weeks, congenital or acquired neuromuscular disorders, congenital or acquired paralysis of the diaphragm, severe traumatic brain injury (i.e., Glasgow Coma Score < 8), uncorrected congenital heart disorder, chronic lung disease and severe pulmonary hypertension. Patients with endotracheal tube (ETT) leakage > 18% were also excluded.

### Ventilator protocol

Prior to enrolment, subjects were ventilated with the AVEA^®^ ventilator (Vyaire, Mettawa, III, USA) in supine position using a time-cycled, pressure limited ventilation mode. This was either in PC-continuous mandatory ventilation [PC-CMV] mode (in our cohort PC assist/control [A/C]) or in a PC-IMV mode (in our cohort PC synchronized intermittent mandatory ventilation [SIMV]) with PS. Choice for PC-CMV or PC-IMV + PS was dictated by patient age (usually, in children < 1 year of age we use PC A/C). Irrespective of mode, an expiratory Vt 5–7 ml/kg actual bodyweight (as there was no obesity in the patient cohort) was targeted and VTe was measured at the Y-piece of the patient circuit (VarFlex™, Vyaire, Mettawa, Ill, USA). Peak inspiratory pressures (PIP) were aimed at < 28 cmH_2_O (< 32 cmH_2_O when there was an increased chest wall elastance). Fraction inspired oxygen was targeted at SpO_2_ of 92–97%. Flow trigger was set between 0.5 and 1.0 L/min. A heat moisture exchanger (Gibeck, Teleflex Medical, Vianen, The Netherlands) was in situ between the patient circuit and the endotracheal tube (ETT) (KimVent, Microcuff Endotracheal Tube, Paediatrics, Roswell, USA).

All patients are routinely instrumented with a catheter to measure the esophageal pressure (Pes) (Avea SmartCath 6 or 8 Fr, Vyaire, Mettawa, III, USA). Correct positioning was visually confirmed by checking for pressure deflections during spontaneous breathing and/or by a chest radiograph that was done for other indications [16].

### Randomization protocol

Baseline defined the ventilator mode and settings that the subject was on before randomization. Subjects were randomized to one of two groups (A and B), defining the order of the weaning approaches tested. Subjects randomized to group A were on CPAP + PS with the level of PS equal to the set pressure above PEEP (PAP) that the subject was on before randomization first, and subsequently to PC-A/C with the ventilator rate set at 25% of baseline. Subjects randomized to group B were on PC-A/C with the ventilator rate set at 25% of baseline first, and subsequently to CPAP + PS.

### Measurement protocol

After obtaining informed consent and enrolment, age appropriate respiratory inductance plethysmography (RIP) bands (Viasys, Healthcare, Respiband Plus, Hoechberg, Germany) were placed circumferentially around the patient’s chest and abdomen. For calibration, the ETT was occluded at the end of an exhalation during a stable breathing for 3–5 consecutive breaths [12, 17]. The esophageal catheter was connected to a BiCore II pulmonary monitor (CareFusion, Houten, The Netherlands) with a sampling frequency of 200 Hz. Then, the esophageal balloon volume was titrated up to a maximum of 1.25 ml H_2_O (pediatric balloon) or 2.5 ml H_2_O (adult balloon). Optimal balloon volume was achieved by titrating volume and graphically depicting the maximum amplitude of the Pes curve (∆Pes).

**Fig. 1:**
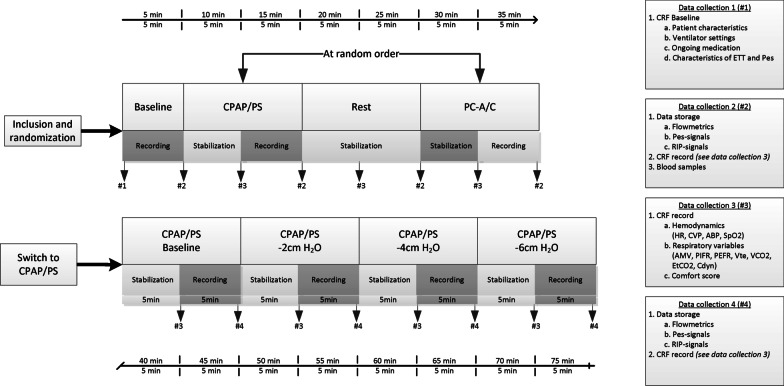
Study design of the different data collection moments during the two different weaning strategies (top figure) and the three step downgrading of pressure support (bottom figure). CRF = case record file, Pes = esophageal pressure, RIP = respiratory inductance plethysmography

Baseline recordings were obtained during 5 min of stable breathing with the ventilator settings the subject was on before randomization. Subsequently, the subject was placed on the ventilator mode and settings according to the randomization outcome. After 5 min of stabilisation, data was then recorded for 5 min. Thereafter, the subject was placed on the baseline ventilator mode and settings for 10 min and then on the ventilator mode and settings according to the randomization outcome. After 5 min of stabilisation, data was then recorded for 5 min. In a second series of measurements, each patient had the level of PS reduced by 2 cmH_2_O on three consecutive steps. Each step consisted of 5 min of stabilization followed by 5 min of recordings (Fig. 1).

Data collection included respiratory rate (RR), heart rate (HR), central venous pressure (CVP), mean arterial blood pressure (ABP), transcutaneous measured oxygen saturation (SpO_2_), minute volume (AMV), expired tidal volume (VTe), end-tidal CO_2_ (EtCO_2_), inspiratory pressures, PEEP, FiO_2_, inspiratory time (Tinsp), mean airway pressure (Pmean) and set flow trigger. Patient comfort was assessed by calculating the Comfort B score [18]. If patients had an indwelling arterial line, blood samples were drawn to determine arterial partial pressure of CO_2_ (PaCO_2_) and O_2_ (PaO_2_). For characterization of the cohort, gender, age, weight, 24-h Pediatric RISk of Mortality (PRISM) III score, admission diagnosis, ETT-size were collected in the database [19]. Respiratory terminology was used based on the Chatburn classification [20].

### Data analysis

Patient inspiratory breathing effort was primary assessed by WOB_CAMPBELL_. Secondary outcomes included PRP, PTP, ∆Pes and the RIP phase angle. Pes and RIP data was analyzed using a custom-build software program (Polybench, Applied Biosignals, Weener, Germany). Pes and RIP signals were first offline reviewed for artifacts (i.e., pressure swings due to esophageal spasms, coughing or body movement) and signal quality. We then selected 30 consecutive, stable breaths and manually placed markers in the RIP and Pes signal to indicate the onset and end of inspiration. WOB_CAMPBELL_ was calculated as the integral of the Pes over the volume displaced during one inhalation [21]. ∆Pes represented the amplitude of inspiratory tidal Pes swings. PTP was calculated by the integral of the Pes signal over time during inspiration multiplied by respiratory rate, and PRP by ∆Pes multiplied by the respiratory rate. The phase angle was calculated from the RIP tracings as described previously [22]. The rapid shallow breathing index (RSBI) was calculated by dividing Vte-exp by the respiratory rate.

### Statistical analysis

Data was assessed for normality using the Kolmogorov–Smirnov test. Descriptive data were expressed as median (interquartile range), percentage (%) or mean (± SD) of total. The Wilcoxon signed rank test was used to detect differences between study time points. By using a generalized, linear mixed model the correlation between WOB_CAMPBELL_ and multiple parameters was studied. Statistical analysis was performed using SPSS v23 (IBM, Armon, NY, USA)*. p* values < 0.05 were considered statistically significant.

## Results

Thirty-six subjects were included (66.7% male) with an overall median age of 4.4 (IQR 1.5–11.9) months and weight 6.5 (IQR 4.6–9.9) kg. Forty-two out of 252 data samples were excluded due to poor quality (Additional file 1: Fig. S1). Patient characteristics were comparable between group A and B (Table 1). Almost all patients were admitted with primary respiratory failure (94.4%). Twenty-seven subjects (75%) had received neuromuscular blockage (NMBA) for a median time of 33.8 (IQR 15.1–41.5) hours. They were discontinued 43.5 (IQR 26.7–71.4) hours before randomization (Additional file 3: Table S1). Baseline ventilator settings before enrolment for the whole cohort was PEEP 6 (IQR 5–6) cmH_2_O, PS 14 (IQR 12–16) cmH_2_O and FiO_2_ 0.30 (IQR 0.26–0.39) (Table 2). Subjects were ventilated for 4.92 (IQR 3.4–7.0) days before enrolment; median time to extubation after enrolment was 23.0 (17.8–44.6) h. Extubation failure (reintubation < 48 h) occurred in 3 patients (8.3%) due to upper airway obstruction (n = 2) or clinically judged excessive work of breathing (n = 1).

**Table 1:** Characteristics of the cohort

	Randomisation group		*P–*value
	A	B	
Number of patients	18	18	
Male (%)	61.1	72.2	0.584
Age (years)	0.56 (0.23–1.34)	0.23 (0.11–0.56)	0.091
0–3 months (%)	27.8	55.6	
3–6 months (%)	22.2	11.1	
6–12 months (%)	11.1	22.2	
1–2 years (%)	27.8	5.6	
2–5 years (%)	11.1	5.6	
Weight (kg)	9.05 (5.15–10.50)	5.40 (4.08–7.07)	0.075
PRISM III (24 h) score	3.00 (2.00–6.00)	3.00 (0.75–4.00)	0.161
PIM II (24 h) score	−4.55 (− 4.67 to − 4.08)	− 4.24 (− 4.74 to − 3.83)	0.584
Admission diagnosis (*n*)			
Respiratory	17	17	1.000
Postoperative	1	1	
Respiratory disease (%)			
Healthy lungs	5.6	5.6	0.539
Obstructive disease	11.1	16.7	
Restrictive disease	22.2	5.6	
Obstructive + restrictive disease	61.1	72.2	
Duration of mechanical ventilation (days)	3.88 (2.66–6.46)	5.94 (3.92–7.83)	0.054
HFO ventilation (%)	44.4	50.0	0.791
HFO ventilation duration (days)	2.15 (1.05–2.94)	2.67 (1.98–4.06)	0.139
Length of PICU stay (days)	5.83 (3.46–8.53)	7.31 (5.11–10.44)	0.085
Extubation outcome			
Reintubation < 48 h (%)	5.6	11.1	0.791
UAO (*n*)	1	1	
Excessive WOB (*n*)	–	1	

Data are shown as number (% of total) or median (interquartile range)

**Table 2:** Patient vital parameters

	Baseline		CPAP/PS	PC-A/C	Baseline	PS -2cmH_2_O	PS -4cmH_2_O	PS -6cmH_2_O
Clinical parameters								
Comfort score	12 (11–15)		11 (11–14)	12 (11–13)	11 (11–14)	11 (11–14)	11 (11–14)	11 (11–14)
Heart rate (beats/min)	138 (123–149)		135 (122–149)	138 (116–150)	134 (117–144)	134 (119–142)	130 (114–149)	133 (117–150)
Peripheral saturation (%)	98 (95–99)		97 (95–98)	98 (96–98)	97 (95–98)	97 (95–98)	96 (94–98)	97 (96 -100)
Respiratory rate (/min)	37 (26–48)		35 (22–48)	32 (23–43)*	37 (21–48)	36 (23–53)*	37 (30–53)*	35 (27–53)
Respiratory parameters								
Expired tidal volume (ml)	41.1 (27.9–82.9)	41.9 (26.2–85.7)		43.8 (26.1–88.2)	41.1 (24.8–79.9)	38.2 (22.2–69.6)*	34.6 (23.7–74.8)*	36.5 (28.2–73.4)
End tidal CO_2_ (mmHg)	49.4 (45.9–54.5)	49.05 (43.7–54.9)		48.8 (45.1–53.4)	49.7 (44.8–52.5)	49.3 (45.6–53.5)	49.4 (44.7–56.3)	53.0 (46.7–55.8)*
T_insp_	0.50 (0.43–0.67)	0.52 (0.39–0.69)		0.63 (0.44–0.73)*	0.52 (0.40–0.76)	0.49 (0.38–0.68)*	0.51 (0.41–0.63)	0.53 (0.43–0.69)
RSBI	0.88 (0.33–1.64)	0.78 ( 0.31–1.70)		0.61 (0.29–1.50)*	0.80 (0.28–1.74)	1.00 (0.33–2.09)*	1.10 (0.40–2.09)	0.88 (0.36–2.07)*
Ventilator settings								
Fraction inspired oxygen (%)	30 (26–39)		30 (26–39)	30 (26–39)	30 (25–40)	30 (27–40)	30 (27–40)	30 (27–40)
PEEP (cmH_2_O)	6 (5–6)		6 (5–6)	6 (5–6)	6 (5–6)	6 (5–6)	6 (5–6)	6 (5–6)
PS (cmH_2_O)^1^	14 (12–16)		14 (12–16)	14 (12–16)	14 (12–16)	12 (10–14)	10 (8–12)	8 (6–10)
Metrics of oxygenation and ventilation								
PaO_2_ (mmHg)	76.51 (66.68–87.76)		74.93 (66.54–85.89)	76.51 (67.21–86.26)	No blood samples withdrawn			
PaCO_2_ (mmHg)	50.10 (56.25–46.50)		50.18 (45.00–5.28)	51.75 (47.25–54.98)				
Oxygenation index	4.92 (3.79–6.03)		4.33 (3.72–5.82)	4.63 (3.79–5.74)				
PF ratio	257 (181–295)		228 (185–278)	249 (192–287)				

Data is compared to the baseline or the previous step in the stepwise reduction of the amount of pressure support. No blood samples were withdrawn during the downgrading of pressure support. Data is shown as median (IQR). Statistic test used is the Wilcoxon signed rank test. *p < 0.05

^1^Set pressure support or the applied pressure above PEEP when on pressure regulated ventilation

### Patient effort during CSV and PC–A/C

**Fig. 2:**
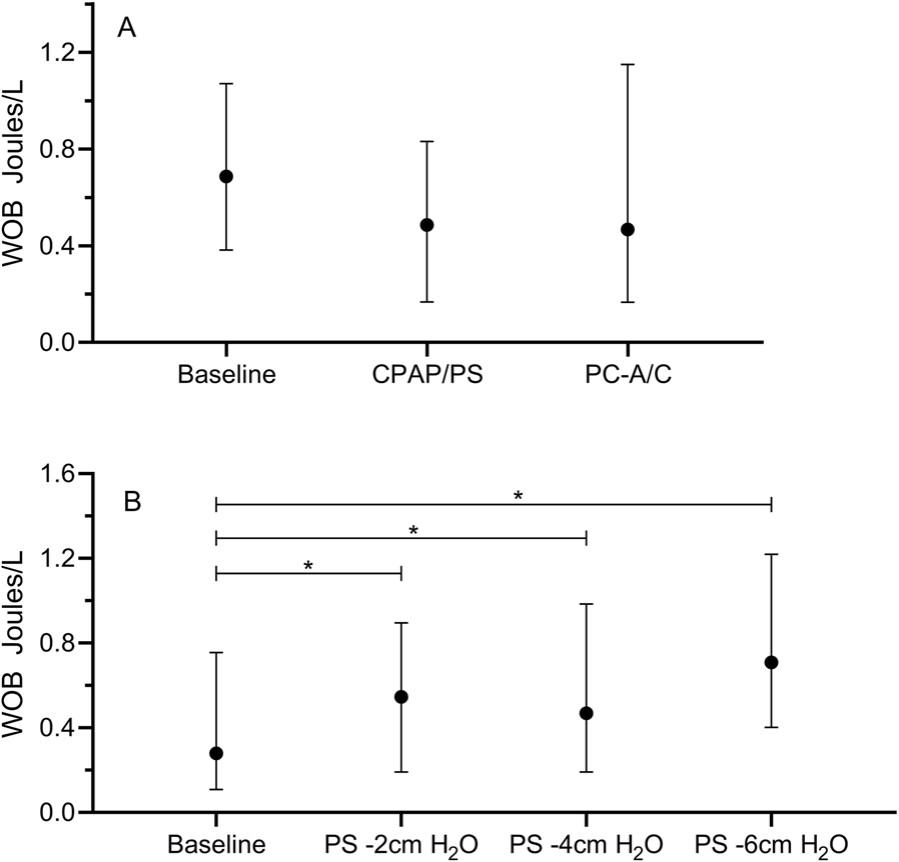
The work of breathing calculated through the gold standard, the Campbell diagram (Joules/L). **a** shows the work of breathing during the different weaning strategies. **b** shows the work of breathing during downtapering of pressure support. *p < 0.05

Median WOB_CAMPBELL_ during baseline recording was 0.67 (IQR 0.38–1.07) Joules/L and decreased to 0.49 (IQR 0.17–0.83) for CPAP/PS and 0.47 (IQR 0.17–1.15) Joules/L for PC–A/C (Fig. 2A). Except for respiratory rate which was significantly higher when patients were in CPAP + PS, no other differences in clinical parameters were observed (Table 2). The Comfort B score was similar between CPAP + PS and PC–A/C.

**Fig. 3:**
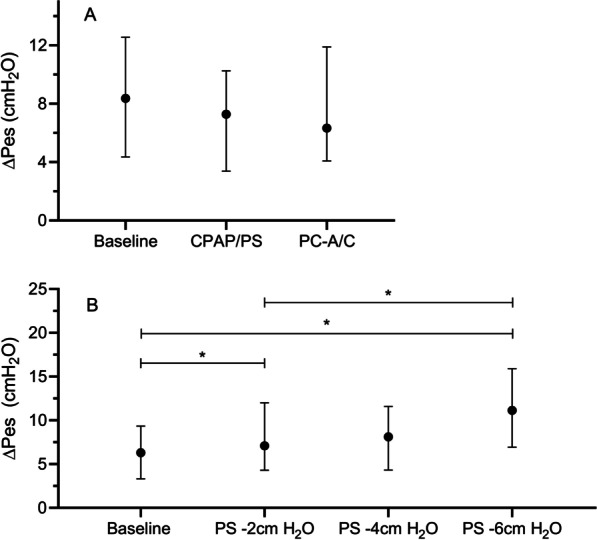
The work of breathing calculated through measuring the difference in esophageal pressure (∆Pes) in cmH_2_O. **a** shows the ∆Pes during the different weaning strategies. **b** shows the ∆Pes during downtapering of pressure support. *p < 0.05

Similar observations regarding comparable patient effort were found in PRP (baseline 296 (IQR 181–445), CPAP + PS 212 (IQR 119–417) and PC–A/C 213 (IQR 140–320) cmH_2_O/min) and PTP (baseline 138 (IQR 68–195), CPAP + PS 105 (IQR 54–170), and PC–A/C 114 (IQR 61–155) cmH_2_O*s/sec) (Additional file 2: Fig. S2). ∆Pes decreased from baseline 8.37 (IQR 4.36–12.56) cmH_2_O to 7.28 (IQR 3.39–10.25) cmH_2_O during CPAP + PS and 6.33 (IQR 4.08–11.89) cmH_2_O during PC-A/C (Fig. 3A). The phase angle was higher during PC–A/C (28.7 (IQR 12.7–42.3), although this did not reach statistical significance when compared to baseline [21.1 (IQR 8.1–42.3)] or during CPAP + PS [25.8 (IQR 1.7–38.6)].

### Patient effort during PS titration

We observed a significant increase in WOB_CAMPBELL_ from baseline [0.28 (IQR 0.11–0.76)] to 0.71 (IQR 0.40–1.22) Joules/L) when PS was decreased by 6 cmH_2_O (Fig. 2B). EtCO_2_ significantly increased, whereas respiratory rate, expiratory Vt (mL/kg) and the RSBI index did not change during the downwards PS titration (Table 2). Similarly, PRP and PTP significantly increased during the downwards PS titration, with PRP increasing to 390 (IQR 231–608) cmH_2_O/min and PTP to 173 (IQR 112–289) cmH_2_O*s/min at PS -6 cmH_2_O. (Additional file 2: Fig. S2) ∆Pes showed a (significant) stepwise increase from 6.31 (IQR 3.33–9.35) cmH_2_O during baseline recordings to 11.14 (IQR 6.92–15.90) cmH_2_O at PS -6 cmH_2_O. (Fig. 3B)The phase angle did not change.

In a correlation analysis, we did not find a significant association between WOB_CAMPBELL_ and duration of MV prior to enrollment, use of high-frequency oscillatory ventilation, ETT size, extubation outcome*,* or NMBA use or time between discontinuation and study measurements.

## Discussion

We have demonstrated in this physiology study that using a continuous spontaneous ventilation mode in pediatric patients resolving from respiratory failure did not lead to increased patient effort compared with an CMV mode. Decreasing PS resulted in a statistically significant, but clinically acceptable increase in patient inspiratory effort. These data may contribute to a better understanding of the patient effort during pediatric ventilation liberation.

To our best of knowledge, this is one of the first studies that compared two different ventilation liberation approaches in children recovering from acute respiratory failure by evaluating patient effort according to the golden standard (i.e., Campbell diagram) [21]. We did not detect clinical relevant differences in patient effort between CPAP/PS and PC–A/C. Observed values for WOB_CAMPBELL_ and PRP and phase angle were in line with previous reported values in children [15, 23–25]. This means that weaning patients in a CSV mode does not lead to increased patient effort. In fact, the PRP values in our study were lower compared with the PRP values reported by Khemani et al. in extubated, spontaneously breathing children [15]. This may suggest that even lower levels of support can be used.

We did observe higher baseline values in WOB_CAMPBELL_, PTP, PRP and ∆Pes than during stable, quiet breathing in CPAP/PS or PC–A/C. This may be explained by the fact that subjects had to be instrumented prior to study measurement which may have caused patient discomfort leading to a temporarily increase in respiratory rate and larger esophageal pressure swings rather than reflecting true increased patient effort, especially since at baseline there was no reduction in ventilator rate or inspiratory pressures. Increases in respiratory rate are easily picked up by PTP and PRP, thus potentially explaining our observations [26].

In our study, we found that patient effort during inspiration increased when PS was decreased, although the clinical relevance of this increase can be questioned. PRP increased, but reached levels that are comparable with the PRP values reported by Khemani et al. [15]. Nonetheless, our data confirms that neither approach do lead to increased patient effort and that a mode in which the patient is more responsible for respiratory homeostasis appears to be at least non-inferior. Since our study was not designed to test superiority or inferiority of CPAP + PS versus PC-A/C with reduced ventilator breath rate, it could be argued that the next step would be to design a randomized controlled trial exploring if weaning and ventilation time can be shortened by one approach or the other.

Our findings also fuel the debate of how much pressure support must be given during pediatric ventilation liberation. It is common practice in pediatrics to add a minimum amount of PS because of the presumed increased resistances of especially smaller endotracheal tubes and thus the fear of increasing the imposed work of breathing (WOB_imp)_, which is the work the patient has to generate to overcome the resistance of the patient circuit and the ETT. In passively breathing patients, this work is done by the ventilator and is added to the work the ventilator has to generate to inflate the lungs [9, 27, 28]. Under spontaneous breathing, the patients have to generate this work, but in ventilator modes that allow spontaneous breaths in-between mandatory breaths, the work by the ventilator during these mandatory breaths may have affected the measured patient effort. Nonetheless, the findings from our present study support previous work from us and others, in which we showed both in a bench and in a clinical study that there was no difference in WOB_imp_ between smaller and larger bigger ETT sizes [29, 30]. Therefore, probably not only during extubation readiness testing but also earlier on during pediatric ventilation liberation it appears to be appropriate to use a lower level of PS when assessing patient effort and that spontaneous breathing trials can be performed without added PS. Setting more PS than actually needed has been shown to overestimate extubation readiness in children [31].

Some limitations of this study need to be addressed. First, it was a single-center study, albeit that it included a homogenous study population, thereby potentially limiting the generalizability although we think this is of no concern for a physiology study such as ours. Second, the 10 min duration for the measurements was arbitrarily chosen as others also have done [13, 15]. Nevertheless, this does not rule out that the period was too short to detect clinically meaningful changes. It may be surmised that a longer duration on each approach could have led to increasing fatigue and different results. Third, the decision to start weaning was at the discretion of the attending physician and not protocolized, making it subject to practice variability and that subjects may have difference in baseline efforts of breathing. Reassuringly, we did not find a significant correlation between duration of ventilation prior to enrolment and indices of patient effort of breathing.

## Conclusion

In children recovering from acute respiratory failure and who are ready to be weaned from the ventilator, effort of breathing was comparable between CPAP + PS and PC-A/C with a reduced ventilator breath rate. Reducing PS did not lead to clinically unacceptable effort of breathing. Our study findings provide helpful insights into optimizing the weaning strategy in ventilated children.

## Supplementary Information

**Additional file 1: Figure S1.** Flow diagram of the study. Pes = esophageal pressure, WOB = work of breathing.

**Additional file 2: Figure S2.** The work of breathing calculated through the pressure–rate-product (PRP) and pressure–time-product (PTP). Figure 3a shows the work of breathing during the different weaning strategies. Figure 3b shows the work of breathing during downtapering of pressure support. *p < 0.05

**Additional file 3: Table S1.** Ventilator and treatment characteristics of the cohort. Data is shown as median (IQR). (1) Ventilation mode before enrollment. (2) Time between stopping neuromuscular blockage and start of inclusion.

## Data Availability

The datasets used and/or analysed during the current study are available from the corresponding author on reasonable request.

## Abbreviations

ABP: Mean arterial blood pressure
A/C: Assist/control
AMV: Minute volume
CPAP: Continuous positive airway pressure
CSV: Continuous spontaneous ventilation
CVP: Central venous pressure
ETT: Endotracheal tube
EtCO_2_: End-tidal CO_2_
FiO_2_: Fraction inspired oxygen
HR: Heart rate
IMV: Intermittent mode of ventilation
IQR: Interquartile range
IRB: Institutional review board
MV: Mechanical ventilation
NMBA: Neuromuscular blockage
PaO_2_: Arterial partial pressure of O_2_
PaCO_2_: Arterial partial pressure of CO_2_
PAP: Pressure above PEEP
PC-A/C: Pressure controlled- assist controlled
PC-IMV: Pressure controlled intermittent mode of ventilation
PCV: Pressure controlled mode of ventilation
PEEP: Positive end expiratory pressure
Pes: Esophageal pressure
PICU: Pediatric intensive care unit
PIP: Peak inspiratory pressure
Pmean: Mean airway pressure
PRISM: Pediatric risk of mortality
PRP: Pressure–rate-product
PS: Pressure support
PTP: Pressure–time-product
RIP: Respiratory inductance plethysmography
RR: Respiratory rate
RSBI: Rapid shallow breathing index
SD: Standard deviation
SpO_2_: Transcutaneous measured oxygen saturation
SIMV: Synchronized intermittent mandatory ventilation
Tinsp: Inspiratory time
USA: United States of America
VILI: Ventilator induced lung injury
VTe: Tidal volume
WOB: Work of breathing
WOB_imp_: Imposed work of breathing
∆Pes: Esophageal pressure swing
