# Supplementary figures and images for: Effect of pediatric ventilation weaning technique on work of breathing
Source: Respir Res. 2022 Jul 13;23:184. doi: 10.1186/s12931-022-02106-6 (PMC9281016; doi:10.1186/s12931-022-02106-6)

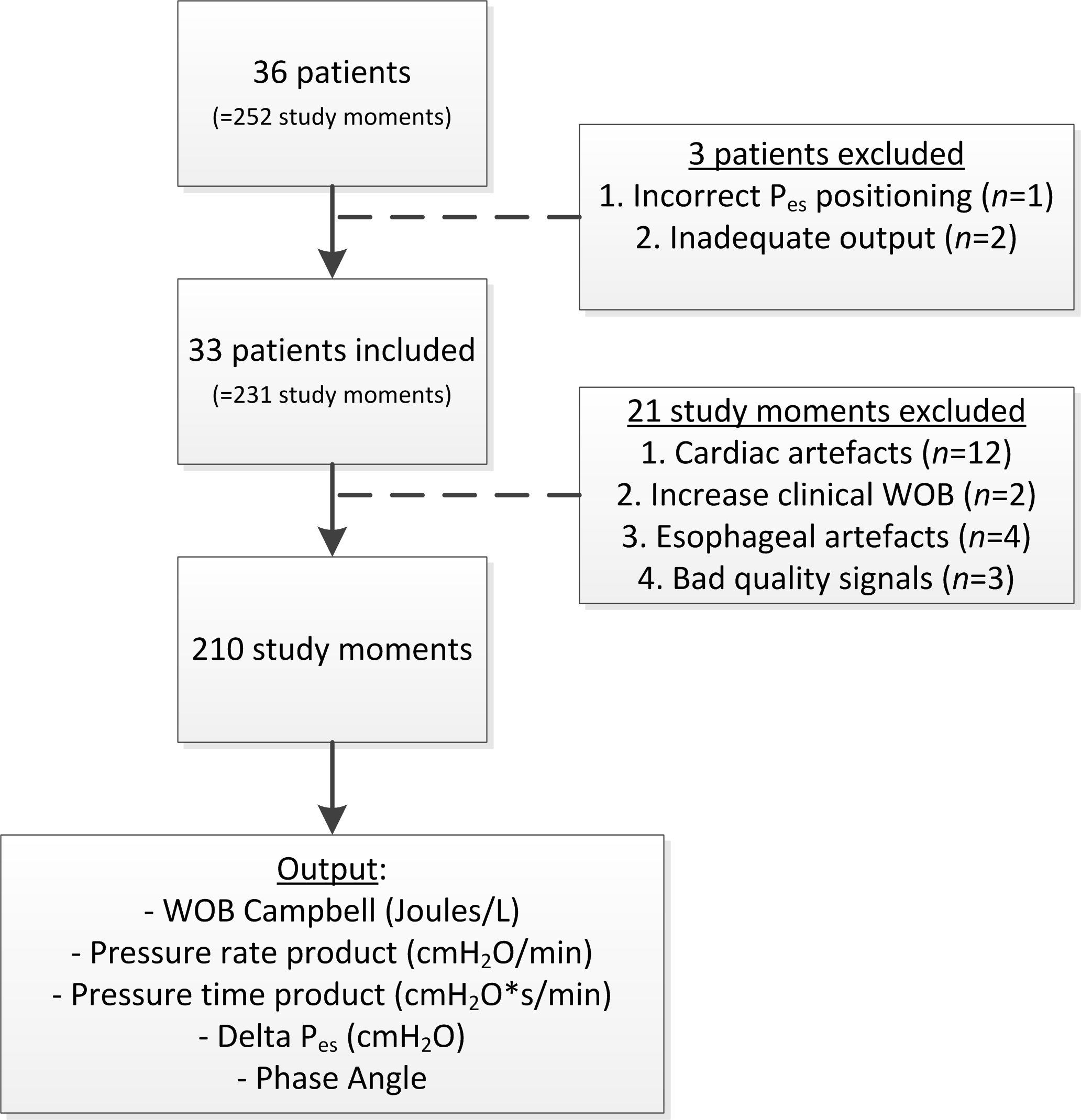

Supplement: Supplementary file 1 — Additional file 1: Figure S1. Flow diagram of the study. Pes = esophageal pressure, WOB = work of breathing. [file 12931_2022_2106_MOESM1_ESM.jpg]
