# Supplementary material for: Effect of pediatric ventilation weaning technique on work of breathing
Source: Respir Res. 2022 Jul 13;23:184. doi: 10.1186/s12931-022-02106-6 (PMC9281016; doi:10.1186/s12931-022-02106-6)
Supplement: Supplementary file 3 — Additional file 3: Table S1. Ventilator and treatment characteristics of the cohort. Data is shown as median (IQR). (1) Ventilation mode before enrollment. (2) Time between stopping neuromuscular blockage and start of inclusion. [file 12931_2022_2106_MOESM3_ESM.docx]

|  | **Randomization group** | |  |
| --- | --- | --- | --- |
|  | **A** | **B** |  |
| **Number of patients** | 18 | 18 |  |
| **Tube Size (*n)***  3.0mm (%)  3.5mm (%)  4.0mm (%)  4.5mm (%) |  |  |  |
|  | 4 (22.2) | 3 (16.7) |  |
|  | 3 (16.7) | 9 (50.0) |  |
|  | 9 (50.0) | 5 (27.8) |  |
|  | 2 (11.1) | 1 (5.6) |  |
| **Tube Location (*n)***  Oral (%)  Nasal (%) |  |  |  |
|  | 7 (38.9) | 6 (33.3) |  |
|  | 11 (61.1) | 12 (66.7) |  |
| **Tube Depth (cm)** | 13.5 (12 - 15.6) | 12.5 (11 – 13.5) |  |
| **Tube Cuff (Y/N) (%)** | 16/2 (88.0/11.1) | 15/3 (83.3/16.7) |  |
| **Ventilator**  **Ventilator mode**  **Ventilator circuit (*n)***  Small (%)  Large (%)  **Ventilation mode^1^ (*n)***  PC-A/C (%)  CPAP/PS (%) |  |  |  |
|  | Pediatric (n=18) | Pediatric (n=18) |  |
|  |  |  |  |
|  | 13 (72.2) | 16 (88.9) |  |
|  | 5 (27.8) | 2 (11.1) |  |
|  |  |  |  |
|  | 16 (88.9) | 17 (94.4) |  |
|  | 2 (11.1) | 1 (5.6) |  |
| **Analgetics**  Morphine (mg/kg/hr)  Fentanyl (ug/kg/hr) |  |  |  |
|  | 10 (5.75 – 10) | 10 (10 – 10) |  |
|  | 1.5 (0.78 – 3.25) | 2 (1 – 2) |  |
| **Sedatives**  Midazolam (mg/kg/hr)  Propofol (mg/kg/hr)  Clonidine (ug/kg/hr) |  |  |  |
|  | 0.1 (0.1 – 0.2) | 0.1 (0.06 – 0.18) |  |
|  | 4 (4 – 4) | 3 (3 – 3) |  |
|  | - | 5 |  |
| **Neuromuscular blockage**  Yes/No (%)  Duration (hrs)  Time between^2^ (hrs) |  |  |  |
|  | 11/7 (61.1/38.9) | 16/2 (88.9/11.1) |  |
|  | 30.0 (15.6 – 51.6) | 35.6 (14.3 – 41.5) |  |
|  | 38.2 (21.7 – 69.7) | 52.4 (29.3 - 74.6) |  |

1. Ventilation mode before enrollment in study
2. Time between stopping of neuromuscular blockage and enrollment in study
